# Trends and causes of mortality in a population-based cohort of HIV-infected adults in Spain: comparison with the general population

**DOI:** 10.1038/s41598-020-65841-0

**Published:** 2020-06-02

**Authors:** Carmen Fontela, Aitziber Aguinaga, Conchi Moreno-Iribas, Jesús Repáraz, María Rivero, María Gracia, Yugo Floristán, Ujué Fresán, Ramón San Miguel, Carmen Ezpeleta, Jesús Castilla

**Affiliations:** 1 Department of Pharmacy, Complejo Hospitalario de Navarra - IdiSNA, Pamplona, Spain; 2 Department of Clinical Microbiology, Complejo Hospitalario de Navarra- IdiSNA, Pamplona, Spain; 3 Instituto de Salud Pública de Navarra - IdiSNA, Pamplona, Spain; 4 Red de Investigación en Servicios de Salud en Enfermedades Crónicas (REDISSEC), Pamplona, Spain; 5 Infectious Diseases Unit, Complejo Hospitalario de Navarra - IdiSNA, Pamplona, Spain; 6 CIBER Epidemiología y Salud Pública (CIBERESP), Pamplona, Spain

**Keywords:** Diseases, Health care, Risk factors

## Abstract

Combination antiretroviral therapy reduces mortality of HIV-infected persons. In Spain, where this therapy is widely available, we aim to evaluate mortality trends and causes of death in HIV-infected adults, and to estimate the excess mortality compared to the general population. From 1999 to 2018 mortality by causes was analyzed in a population-based cohort of adults aged 25 to 74 years diagnosed with HIV infection in Spain. Observed deaths and expected deaths according mortality in the general population of the same sex and age were compared using standardized mortality ratios (SMRs). HIV-infected people increased from 839 in 1999–2003 to 1059 in 2014–2018, median age increased from 37 to 47 years, the annual mortality rate decreased from 33.5 to 20.7 per 1000 person-years and the proportion of HIV-related deaths declined from 64% to 35%. HIV-related mortality declined from 21.4 to 7.3 (p < 0.001), while non-HIV-related mortality remained stable: 12.1 and 13.4 per 1000, respectively. Mortality decreased principally in persons diagnosed with AIDS-defining events. In the last decade, 2009–2018, mortality was still 8.1 times higher among HIV-infected people than in the general population, and even after excluding HIV-related deaths, remained 4.8 times higher. Excess mortality was observed in non-AIDS cancer (SMR = 3.7), cardiovascular disease (SMR = 4.2), respiratory diseases (SMR = 7.9), liver diseases (SMR = 8.8), drug abuse (SMR = 47), suicide (SMR = 5.3) and other external causes (SMR = 6). In conclusion, HIV-related mortality continued to decline, while non-HIV-related mortality remained stable. HIV-infected people maintained important excess mortality. Prevention of HIV infections in the population and promotion of healthy life styles in HIV-infected people must be a priority.

## Introduction

Since the introduction of combined antiretroviral therapies in the second half of the 1990s, morbidity and mortality in HIV-infected persons have decreased steeply^1–3^, and their life expectancy has improved^3–5^. HIV-infected persons may die of HIV-related and non-HIV-related causes. The first ones mark the difference with the general population, but may be averted by early and successful antiretroviral therapy^3^. HIV-related mortality has been associated with the long length of infection, higher age at seroconversion, late HIV diagnosis, incomplete adherence to antiretroviral therapies and use of suboptimal regimens^1,2,6,7^. Risk behaviours related to HIV transmission may have other negative effects in health as drug abuse problems and other sexually or blood transmitted infections as hepatitis B or C that could increase non-HIV-related mortality^8–12^.

Clinical cohorts of HIV-infected patients have described important reduction in mortality^8–12^, but they may not be representative of the overall mortality in the HIV-infected population, because HIV-infections diagnosed before the combined antiretroviral therapy availability, patients deceased before starting of clinical follow-up, those who refuse treatments or who do not contact the healthcare system are usually excluded.

In the first years with availability of effective antiretroviral therapies, a considerable excess mortality was still reported in HIV-infected persons^13^. Since then, relevant clinical advances has been introduced: the initiation of treatment turned from a threshold of CD4 count to as soon as possible after diagnosis^6,14,15^, the progressive introduction of safer antiretroviral combinations^16^, and direct acting antiviral regimens for hepatitis C virus (HCV) co-infected patients^17^. All these interventions are expected to further reduce the excess mortality in people living with HIV-infection. Therefore, we aimed to describe recent changes in mortality and causes of death in a population-based cohort of persons diagnosed with HIV infection, and to compare with mortality in the general population of the same age and sex and living in the same region.

## Results

### Characteristics of the cohort

From 1999–2003 to 2014–2018 the number of prevalent cases diagnosed with HIV infection increased from 839 to 1059. The proportion of women remained around one third. HIV infections related to injecting drug use decreased from 71.2% to 43.1%, and those in men who had sex with men increased from 6.3% to 20.0%. The median age increased from 37 to 47 years, and the time since the diagnosis of HIV from 9 to 14 years. The proportion of people born out of Spain increased from 3.6% to 19.5%, and patients who have started antiviral therapy increased from 72.1% to 87.9%. The percentage of HCV-RNA positive patients declined from 29.9% to 25.4%, and most of them (93.7%) had history of injecting drug use. HIV-infected people in follow-up who had been diagnosed with AIDS decreased from 35.3% to 27.0% (Table 1).

**Table 1:** Characteristics of the cohort of people diagnosed with HIV infection by study period.

	1999–2003	2004–2008	2009–2013	2014–2018
HIV-infected persons in follow-up, n	839	881	964	1059
Sex, n (%)				
Male	568 (67.7)	584 (66.3)	637 (66.1)	709 (66.9)
Female	271 (32.3)	297 (33.7)	327 (33.9)	350 (33.1)
Transmission category, n (%)				
Injecting drug use	597 (71.2)	550 (62.4)	499 (51.8)	456 (43.1)
Men who had sex with men	53 (6.3)	80 (9.1)	133 (13.8)	212 (20.0)
Heterosexual transmission	166 (19.8)	220 (25.0)	285 (29.6)	340 (32.1)
Other risk or unknown	23 (2.7)	31 (3.5)	47 (4.9)	51 (4.8)
Age, years, median [interquartil range]^a^	37 [34–41]	41 [37–44]	43 [39–47]	47 [40–52]
Time since HIV diagnosis, median in years [interquartil range]^a^	9 [4–12]	11 [6–15]	13 [4–18]	14 [4–22]
Year of HIV diagnosis, median [interquartil range]	1992 [1988–1996]	1993 [1990–1999]	1996 [1991–2004]	1999 [1992–2010]
AIDS-defining event diagnosis, n (%)	296 (35.3)	307 (34.8)	291 (30.2)	286 (27.0)
Anti-HCV positive, n (%)	322 (38.4)	368 (41.8)	383 (39.7)	405 (38.2)
HCV-RNA positive ever, n (%)	251 (29.9)	264 (29.9)	268 (27.8)	270 (25.4)
Born out of Spain, n (%)	30 (3.6)	84 (9.5)	145 (15.0)	207 (19.5)
Antiretroviral treatment, n (%)	605 (72.1)	660 (74.9)	745 (77.3)	931 (87.9)
Person-years of follow-up	3555	3826	4172	4693
Deaths, n (%)	119 (14.2)	101 (11.5)	89 (9.2)	97 (9.2)
Mortality rate per 1000 person-years	33.5	26.4	21.3	20.7

^a^Data referred to the middle of the period.

HCV: hepatitis C virus.

### Trends in mortality

All-cause crude mortality fell by 38% from 33.5 in 1999–2003 to 20.7 per 1000 person-years (PY) in 2014–2018. Crude mortality declined by 3% annually and mortality adjusted for sex, age, foreign-born and transmission category declined by 5% (adjusted rate ratio [aRR] = 0.95; p < 0.001). In 1999–2003, mortality rate was higher in males than females (38.8 vs 22.9 per 1000 PY; p = 0.013); however, the more pronounced decline in mortality in males (aRR = 0.95, p < 0.001) than in females (aRR = 0.98, p = 0.323) resulted in similar mortality rates in 2014–2018 (21.0 and 20.1 per 1000 PY, respectively; p = 0.859). Declines in adjusted mortality rates were similar regardless the age group, history of injecting drug use or the foreign-born. All-cause mortality decreased regardless if HIV diagnosis had been before or in or after 1997 (aRR = 0.95, p = 0.0068 and aRR = 0.96, p = 0.013, respectively) (Table 2).

**Table 2:** Number of deaths, annual average crude mortality rate per 1000 person-years, and crude and adjusted annual mortality trend in the cohort of people diagnosed with HIV infection.

	1999–2003		2004–2008		2009–2013		2014–2018		Unadjusted trend^a^		Adjusted trend^a^	
	N	Rate	N	Rate	N	Rate	N	Rate	RR	p-value	aRR	p-value
Sex												
Male	92	38.8	77	30.7	64	23.8	65	21.0	0.96	<0.001	0.95	<0.001
Female	27	22.9	24	18.3	25	16.8	32	20.1	0.99	0.627	0.98	0.326
Age, years												
<45 years	93	29.2	70	23.5	41	17.6	20	11.9	0.95	<0.001	0.96	0.016
≥45 years	26	71.0	31	36.8	48	26.1	77	25.6	0.94	<0001	0.94	<0.001
History of injecting drug use												
Yes	90	33.8	74	29.7	57	24.9	58	27.5	0.98	0.115	0.96	0.004
No	29	32.6	27	20.3	32	17.0	39	15.1	0.95	0.003	0.95	0.002
Country of birth												
Spain	117	33.7	98	27.7	84	23.4	87	22.9	0.97	0.003	0.95	<0.001
Other	2	24.4	3	10.4	5	8.6	10	11.1	0.98	0.636	0.96	0.445
Year of HIV diagnosis												
Before 1997	96	32.7	80	30.2	62	26.3	56	26.7	0.95	0.006	0.95	0.007
1997 or after	23	37.1	21	17.9	27	14.9	41	15.8	0.98	0.142	0.96	0.001
Total	119	33.5	101	26.4	89	21.4	97	20.7	0.97	<0.001	0.95	<0.001

^a^Average annual trend unadjusted and adjusted for sex, age group (25–34; 35–44, 45–54, 55–64, 65–74), country of birth (Spain or other) and transmission category (history of injecting drug use, men who had sex with men, heterosexual risk exposure, and other risk or unknown).

RR, rate ratio of trend for annual change in mortality.

HIV-related deaths were responsible for 64% of the total mortality in the cohort in 1999–2003 and decreased to 35% in 2014–2018. HIV-related crude mortality decreased 66%, from 21.4 to 7.2 per 1000 PY, and the adjusted rate decreased 8% annually (aRR = 0.92; p < 0.001). Crude mortality from other causes increased slightly, from 12.1 to 13.5 per 1000 PY, but no statistically significant trend was detected in the crude or adjusted analysis (aRR = 1.00; p = 0.913). Mortality due to drug addiction or overdose decreased from 4.2 to 0.9 per 1000 PY, that supposes an unadjusted annual decline of 11% (unadjusted RR = 0.89; p = 0.001), but this trend partially disappeared in the adjusted analysis (aRR = 0.94; p = 0.183). No statistically significant change was observed in any of the other specific non-HIV-related causes of death (Table 3).

**Table 3:** Number of deaths, annual average crude mortality rate per 1000 person-years, and crude and adjusted annual mortality trend in the cohort of people diagnosed with HIV infection.

	1999–2003		2004–2008		2009–2013		2014–2018		Unadjusted trend^a^		Adjusted trend^a^	
	N	Rate	N	Rate	N	Rate	N	Rate	RR	p-value	aRR	p-value
**All HIV-infected persons**	119	33.5	101	26.4	89	21.4	97	20.7	0.97	<0.001	0.95	<0.001
HIV-related mortality	76	21.4	62	16.2	43	10.3	34	7.2	0.93	<0.001	0.92	<0.001
Non-HIV-related mortality	42	12.1	39	10.0	46	11.1	63	13.5	1.01	0.461	1.00	0.913
Cancer (non-AIDS)	9	2.5	17	4.4	19	4.6	21	4.5	1.03	0.213	1.00	0.950
Cardiovascular disease	6	1.7	3	0.8	4	1.0	13	2.8	1.05	0.166	1.02	0.673
Respiratory disease	0	0.0	3	0.8	3	1.0	4	0.9	1.09	0.182	1.04	0.566
Liver disease	0	0.0	3	0.8	5	1.2	3	0.6	1.07	0.262	1.03	0.669
Drug addiction or overdose	15	4.2	5	1.3	4	0.7	4	0.9	0.89	0.012	0.94	0.183
Suicide	4	1.1	3	0.8	3	0.7	4	0.9	0.98	0.698	1.00	0.931
Other external causes	4	1.1	2	0.5	4	1.0	4	0.9	0.97	0.876	0.97	0.666
All other causes	5	1.4	3	0.8	4	1.0	10	2.1	1.05	0.192	1.05	0.300
**Diagnosis of ADE**	74	71.2	64	54.7	44	36.7	44	36.2	0.95	<0.001	0.94	<0.001
HIV-related mortality	58	55.8	46	39.3	26	21.7	21	17.3	0.92	<0.001	0.91	<0.001
Non-HIV-related mortality	16	15.4	18	15.4	19	15.8	23	18.9	1.01	0.529	1.01	0.595
**Not diagnosis of ADE**	45	17.9	37	13.9	45	15.1	53	15.2	0.99	0.557	0.98	0.281
HIV-related mortality	18	7.2	16	6.0	17	5.7	13	3.7	0.96	0.080	0.96	0.218
Non-HIV-related mortality	27	10.7	21	7.9	27	9.1	40	11.5	1.01	0.564	0.99	0.691

^a^Average annual trend unadjusted and adjusted for sex, age group (25–34; 35–44, 45–54, 55–64, 65–74), country of birth (Spain or other) and transmission category (history of injecting drug use, men who had sex with men, heterosexual risk exposure, and other risk or unknown).

RR, rate ratio of trend for annual change in mortality.

ADE, AIDS-defining events.

In patients who had had diagnosis of AIDS-defining events mortality decreased from 71.2 to 36.2 per 1000 PY (aRR = 0.94; p < 0.001), but in people never diagnosed with AIDS-defining events the decline was minor and not statistically significant, from 17.9 to 15.2 (aRR = 0.98; p = 0.281) (Table 3).

### Comparison with mortality in the general population

In the 1999–2003 period, all-cause mortality in people diagnosed with HIV infection was 20.4 (95% confidence interval [CI]: 17.0–24.3) times higher than in the general population of the same sex and age group, and in 2014–2018 it was still 7.4 (95% CI: 6.0–9.0) times higher. After excluding HIV-related deaths, mortality in HIV-infected people declined from 7.2 to 4.8 times higher than that of the general population in the same periods (Table 4). For all groups of HIV-infected patients analysed, mortality was statistically significant higher than expected from the general population, but in general, these differences declined over time. Standard mortality ratios (SMRs) were higher in females than males, and in people younger than 45 years than in the older ones; however the excess mortality rates were in general higher in males and in the older age group. Although the lowest SMRs were observed in HIV-infected men who had sex with men, in the period 2014–2018 their mortality was still 4.3 times higher than in male general population. Excess mortality rates and SMRs were higher in persons diagnosed with HIV before 1997 than in those diagnosed in or after, but in both cases declined over time. Nevertheless, in the period 2014–2018, mortality in persons diagnosed with HIV infection in 1997 or after still was 6.1 higher than in general population (Table 4).

**Table 4:** Comparison of mortality between HIV-infected subjects and the general population of the same sex and age group, by population group and period, 1999–2018.

Population group and period	Number of deaths	Mortality rate per 1000 PY	Excess mortality rate per 1000 PY (95% CI)^a^	Standardized mortality ratio (95% CI)^a^
**All-cause deaths**				
1999–2003	119	33.5	31.8 (26.4–37.9)	20.4 (17.0–24.3)
2004–2008	101	26.4	24.6 (20.1–29.5)	14.2 (11.7–17.2)
2009–2013	89	21.4	19.0 (15.4–23.4)	9.1 (7.3–11.1)
2014–2018	97	20.7	17.9 (14.5–21.7)	7.4 (6.0–9.0)
**Non-HIV-related deaths**				
1999–2003	42	11.8	10.2 (7.6–13.7)	7.2 (5.3–9.7)
2004–2008	39	10.0	8.4 (5.9–11.2)	5.5 (4.0–7.4)
2009–2013	46	11.1	8.7 (6.5–11.7)	4.7 (3.5–6.2)
2014–2018	63	13.4	10.6 (8.1–13.6)	4.8 (3.7–6.1)
**Male**				
1999–2003	92	38.8	36.6 (29.8–44.3)	18.3 (14.8–22.3)
2004–2008	77	30.7	28.4 (22.8–35.3)	13.0 (10.4–16.2)
2009–2013	64	23.9	20.8 (16.1–26.3)	7.7 (5.9–9.7)
2014–2018	65	21.0	17.4 (1.34–22.0)	5.9 (4.6–7.5)
**Female**				
1999–2003	27	22.8	22.2 (15.9–31.1)	33.2 (22.5–47.5)
2004–2008	24	18.3	17.4 (11.7–24.4)	20.2 (13.2–29.6)
2009–2013	25	16.8	15.8 (10.9–22.4)	17.2 (11.4–25.1)
2014–2018	32	20.1	18.8 (13.2–25.4)	15.1 (10.5–21.1)
**Aged <45 years**				
1999–2003	93	29.2	28.0 (23.0–34.0)	24.8 (20.1–30.2)
2004–2008	70	23.5	22.4 (17.7–27.8)	22.1 (17.4–27.8)
2009–2013	41	17.6	16.7 (12.6–22.3)	20.9 (15.2–28.1)
2014–2018	20	11.9	11.2 (7.3–16.2)	18.1 (11.4–27.4)
**Aged ≥45 years**				
1999–2003	26	71.0	65.3 (32.8–91.3)	12.4 (8.3–18.0)
2004–2008	31	37.0	32.3 (23.2–45.5)	7.9 (5.5–11.1)
2009–2013	48	26.2	21.9 (16.5–29.1)	6.1 (4.6–8.0)
2014–2018	77	25.6	21.6 (17.0–26.8)	6.4 (5.1–7.9)
**History of injecting dug use**^b^				
1999–2003	89	33.4	32.0 (26.1–39.0)	25.2 (20.4–30.9)
2004–2008	74	29.6	28.1 (22.6–35.0)	19.9 (15.8–24.9)
2009–2013	57	24.8	22.7 (17.7–29.2)	11.7 (8.9–15.0)
2014–2018	58	27.4	24.6 (19.2–31.7)	9.9 (7.6–12.7)
**Men who had sex with men**^c^				
1999–2003	3	14.9	12.1 (5.4–27.6)	5.4 (1.4–14.6)
2004–2008	7	22.1	19.4 (10.9–36.8)	8.2 (3.6–16.3)
2009–2013	5	10.1	7.4 (3.3–14.6)	3.7 (1.4–8.3)
2014–2018	10	11.5	8.8 (4.7–15.0)	4.3 (2.2–7.7)
**Heterosexual risk exposure**^b^				
1999–2003	24	40.2	37.6 (25.8–53.8)	15.2 (10.0–22.3)
2004–2008	19	21.5	19.0 (12.1–28.0)	8.5 (5.3–13.0)
2009–2013	22	18.4	15.9 (10.2–22.8)	7.2 (4.6–10.7)
2014–2018	24	16.3	13.5 (8.3–19.3)	5.8 (3.8–8.4)
**HIV diagnosis before 1997**				
1999–2003	96	32.7	31.2 (25.6–37.7)	22.0 (17.9–26.8)
2004–2008	80	30.2	28.4 (22.9–35.1)	17.1 (13.6–21.1)
2009–2013	62	26.3	23.9 (18.7–30.4)	10.9 (8.4–13.8)
2014–2018	56	26.7	23.6 (18.1–30.3)	8.6 (6.5–11.0)
**HIV diagnosis in 1997 or after**				
1999–2003	23	37.1	34.7 (23.5–49.8)	15.3 (9.9–22.5)
2004–2008	21	17.9	15.8 (10.4–23.2)	8.6 (5.5–12.9)
2009–2013	27	14.9	12.6 (8.5–17.7)	6.5 (4.4–9.3)
2014–2018	41	15.8	13.2 (9.7–17.9)	6.1 (4.4–8.2)

^a^Mid-P exact test.

^b^Expected deaths were calculated from the total reference population.

^c^Expected deaths were calculated from the male reference population.

CI, confidence interval; PY, person-years.

In the 2009–2018 decade, mortality in HIV-infected population was statistically significant higher than the expected for all non-HIV-related causes of death considered. The most pronounced SMRs were observed in drug addiction or overdose (46.7 times higher), liver disease (8.8 times higher) and respiratory disease (7.9 times higher); however, in absolute terms, the highest excess mortality rates were observed in non-AIDS cancer (3.3 deaths per 1000 PY) and cardiovascular disease (1.5 per 1000 PY). Excess mortality rates were quite similar in males (19.0 per 1000 PY) and females (17.4 per 1000 PY); however, this resulted in a higher SMR for females (16.1) than for males (6.7). Excess mortality was more pronounced in HIV-infected persons with history of injecting drug use. In this group, all-cause mortality was 11 times higher and non-HIV related mortality was 6.9 times higher than in the general population, while they were 5.7 and 3.1 times higher, respectively, among people without history of injecting drug use (Table 5).

**Table 5:** Comparison of mortality by causes between HIV-infected subjects and the general population of the same sex and age group, 2009–2018.

	Number of deaths	Mortality rate per 1000 PY	Excess mortality rate per 1000 PY (95% CI)^a^	Standardized mortality ratio (95% CI)^a^
**All HIV-infected persons**				
**All-cause** **mortality**	186	21.0	18.4 (15.9–21.4)	8.1 (7.0–9.3)
**Non-HIV-related mortality**	109	12.3	9.7 (8.0–11.9)	4.8 (3.9–5.7)
Cancer (non-AIDS)	40	4.5	3.3 (2.4–4.6)	3.7 (2.7–5.0)
Cardiovascular disease	17	1.9	1.5 (0.9–2.2)	4.2 (2.5–6.5)
Respiratory disease	7	0.8	0.7 (0.4–1.3)	7.9 (3.4–15.5)
Liver disease	8	0.9	0.8 (4.6–14.8)	8.8 (4.1–16.6)
Drug addiction or overdose	8	0.9	0.9 (0.5–1.5)	46.7 (21.7–88.6)
Suicide	7	0.8	0.6 (3.2–1.2)	5.3 (2.3–10.6)
Other external causes	8	0.9	0.8 (0.4–1.3)	6.0 (2.8–11.4)
All other causes	14	1.1	1.2 (0.7–1.9)	4.0 (2.3–6.5)
**Male**				
** All-cause** **mortality**	129	22.3	19.0 (15.8–22.6)	6.7 (5.6–7.9)
** Non-HIV-related mortality**	78	13.5	10.2 (7.9–12.8)	4.0 (3.2–5.0)
Cancer (non-AIDS)	27	4.7	3.2 (2.1–4.7)	3.1 (2.1–4.5)
Cardiovascular disease	12	2.1	1.4 (0.8–2.5)	3.2 (1.7–5.4)
Respiratory disease	5	0.9	0.7 (3.8–15.2)	6.5 (2.4–14.3)
Liver disease	7	1.2	1.1 (0.6–2.0)	8.4 (3.7–16.7)
Drug addiction or overdose	6	1.0	1.0 (0.5–1.8)	38.0 (15.4–79.0)
Suicide	6	1.0	0.9 (0.4–1.5)	5.7 (2.3–11.8)
Other external causes	3	0.5	0.3 (0.1–0.6)	2.5 (0.6–6.7)
All other causes	12	1.4	1.6 (1.0–2.7)	4.1 (2.2–6.9)
**Female**				
**All-cause** **mortality**	57	18.5	17.4 (12.5–22.2)	16.1 (12.3–20.7)
** Non-HIV-related mortality**	31	10.0	8.9 (6.3–12.4)	8.7 (6.1–12.3)
Cancer (non-AIDS)	13	4.2	3.5 (2.0–5.5)	6.2 (3.4–10.3)
Cardiovascular disease	5	1.6	1.5 (0.7–2.8)	16.3 (6.0–36.2)
Respiratory disease	2	0.6	0.6 (0.2–1.2)	17.0 (2.9–56.2)
Liver disease	1	0.3	0.3 (0.1–15.0)	12.0 (0.6–59.1)
Drug addiction or overdose	2	0.6	0.6 (0.2–1.2)	147.7 (24.8–488)
Suicide	1	0.3	0.2 (0.1–7.9)	3.9 (0.2–19.2)
Other external causes	5	1.6	1.6 (0.7–2.8)	44.5 (16.3–98.7)
All other causes	2	0.6	0.5 (0.2–1.2)	3.6 (0.6–12.0)
**History of injecting drug use**				
**All-cause** **mortartality**	117	26.6	24.2 (20.1–28.9)	11.0 (9.1–13.1)
**Non-HIV-related mortality**	52	16.6	14.2 (11.2–17.8)	6.9 (5.4–8.6)
Cancer (non-AIDS)	24	5.5	4.4 (3.0–6.5)	4.9 (3.2–7.2)
Cardiovascular disease	10	2.3	1.8 (1.1–3.3)	5.3 (2.7–9.5)
Respiratory disease	3	0.7	0.6 (0.2–1.3)	8.8 (2.2–24.0)
Liver disease	8	1.8	1.7 (9.4–3.0)	16.8 (7.8–31.9)
Drug addiction or overdose	7	1.6	1.6 (0.8–2.7)	74.1 (32.5–147)
Suicide	5	1.1	1.0 (0.5–2.0)	6.8 (2.5–15.2)
Other external causes	6	1.4	1.2 (0.6–2.3)	9.3 (3.8–19.3)
All other causes	8	1.8	1.5 (0.8–2.7)	5.0 (2.3–9.7)
**No history of injecting drug use**				
**All-cause** **mortality**	71	15.9	13.1 (10.3–16.6)	5.7 (4.5–7.2)
**Non-HIV-related mortality**	39	8.7	6.0 (4.2–8.3)	3.1 (2.3–4.2)
Cancer (non-AIDS)	16	3.6	2.3 (1.4–3.8)	2.7 (1.6–4.3)
Cardiovascular disease	7	1.6	1.1 (0.5–2.0)	3.1 (1.4–6.2)
Respiratory disease	4	0.9	0.8 (0.4–1.6)	7.2 (2.3–17.3)
Liver disease	0	0	0	—
Drug addiction or overdose	1	0.2	0.2 (0.1–1.2)	12.9 (0.6–63.8)
Suicide	2	0.4	0.3 (0.1–0.8)	3.4 (0.6–11.4)
Other external causes	2	0.4	0.3 (0.1–0.8)	2.9 (0.5–9.5)
All other causes	7	1.6	1.1 (0.6–2.3)	3.6 (1.6–7.2)

^a^Mid-P exact test.

CI, confidence interval; PY, person-years.

## Discussion

From 1999 to 2018, the adjusted mortality rate in a population-based cohort of people with a diagnosis of HIV infection declined by 5% annually, following the trend that had begun with the introduction of highly active antiretroviral therapies^3,10^. This trend has contributed to reduce the excess mortality in the HIV-infected population. Nevertheless, in the 2014–2018 period, mortality still was 7.4 times higher than in the general population of the same sex and age. HIV-infected men who had sex with men showed the lowest excess mortality as compared to male general population, probably due to the earlier diagnoses and lower probability of other risk factors and comorbidities than in other transmission categories; however their mortality was still 4.3 times higher than expected in the last period. Although persons diagnosed with HIV infection in 1997 or after, period of use of combined antiretroviral therapy, had lower mortality than those diagnosed before, they still had 6.1 times higher mortality than the general population. These findings suggest that one part of the excess mortality associated to HIV-infection may be very difficult to control even with wide availability of antiretroviral treatments, and that promotion of healthy lifestyles in these patients is also very important. These findings also highlight the great advantage in terms of life expectancy that primary prevention of HIV transmission still has over the antiretroviral treatment.

The excess mortality observed in the current study was greater than that reported from clinical cohorts of HIV-infected patients, which may be explained by selection of patients in this kind of cohorts^8–12^; however, our results are close to those of another population-based study^18–20^.

HIV-related mortality decreased from 2 to 1 out of 3 deaths during the study period. The HIV-related mortality rate declined 8% annually, being the main component of the reduction in excess overall mortality among HIV-infected population. Although highly active antiretroviral therapy was already available before the study period, since then safer antiretroviral drug combinations that favour adherence have been progressively introduced^16^, and initiation of these treatments turned from a threshold based on CD4 count to as soon as possible after diagnosis may have contributed to maintain the reduction in HIV-related mortality^6,14,15^. The main reduction in mortality was observed in patients with diagnosis of AIDS-defining events, in whom the risk of mortality^21^ and the potential benefit by a successful antiretroviral therapy are higher. However, mortality reduction was small or null in HIV-infected persons without prior AIDS-defining events, since mortality among them is mainly related to other comorbidities and risk factors, and its prevention requires other kind of preventive and healthcare interventions.

Non-HIV-related mortality remained stable during the study period, with an important excess as compared to the general population. Excess mortality in relative terms (SMR) was more pronounced in drug addiction or overdose and liver disease, since these causes were strongly associated to history of injecting drug use, which was present in many of HIV-infected people^11,13^. Nevertheless, the highest excess mortality rates were observed in cancer and cardiovascular disease, since they were also leader causes of death in HIV-infected population as they are in the general population. This finding could be explained by the higher frequency of tobacco, alcohol and recreational drug consumption, the faster aging and the premature presentation of comorbidities in HIV-infected people^22^.

History of injecting drug use was associated to higher mortality by most causes. It was present in the majority of deaths by drug overdoses or addiction, was a risk factor for coinfections by hepatitis B and C viruses and was frequently associated to injuries, suicide and to unhealthy life styles as alcohol abuse and smoking^23,24^.

During the study period, excess mortality rates declined more in HIV-infected males than in females, and by the last study period the differences by sexes had disappeared. Females presented higher excess mortality in relative terms, since in the general population women have lower mortality than men. This is partly explained because women assume less risk and take more care of their health, which may not be true in HIV-infected females, who have been associated with situations of disadvantage for the care of their health^24–26^.

Since excess mortality rates were higher in people older than 45 years, the aging of the cohort and the premature presentation of comorbidities^22^ favor to maintain the excess mortality. However the higher SMRs in people under 45 years of age indicate the important relative impact of HIV infection on mortality since young ages.

In the studied cohort, people with history of injecting drug use predominated, although there was a progressive increase in the proportion of people probably infected by sexual transmission, coinciding with the epidemiological change described in Spain^27^. Median age and time since the diagnosis of HIV increased due to the aging of the cohort. The percentage of patients who had been diagnosed with AIDS-defining events decreased throughout the study, due to the lower incorporation of new cases and the higher mortality associated with this situation^18–21,23,28,29^. Although the proportion of people born out of Spain increased, they did not show higher mortality than Spain-born people as it has been reported in other countries^26^. This can be due to the less time of HIV-infection and the similar access to the HIV-diagnosis and treatment.

The average percentage of patients who had started antiretroviral therapy increased up to 87.9% in the 2014–2018 period, when the indication of this therapy was extended to all HIV diagnosed patients. Patients who had not started therapy may be explained by the transition process from old to new guidelines, patients deceased before starting of clinical follow-up, those who refuse treatments or who do not contact the healthcare system. This study was conducted in a country with universal and free of charge health-care coverage for the entire resident population, including HIV diagnosis and antiretroviral treatment. This fact discards socioeconomic reasons for not receiving medical follow-up and antiretroviral treatment; and therefore, differences in mortality should be explained by other causes. In Spain, it has been estimated that 29% and 48% of HIV diagnoses were done in patients with advanced disease and late diagnosis, respectively^30^. These figures show a potential for improvement according the 2020 UNAIDS strategy, which goals are 90% of HIV-infected people diagnosed, 90% of them receiving antiretrovirals, and 90% of those in treatment achieving viral suppression^31^.

The present work has some limitations. This descriptive population-based study is not adequate to evaluate survival or to conclude about causal factors since many participants did not enter at the seroconversion or HIV diagnosis, and variables such as antiretroviral therapy, viral load, CD4 cell count, smoking status and drug consumption were not monitored during the follow-up. The study has been carried out in a single region and the study size could be small for some analyses. The analysis was limited to the population with stable residence, and non-resident patients were not included. It has been described that populations with high mobility could have worse follow-up of their health problems^32^. Losses of follow-up are a relevant source of bias in cohort studies; however, people were included only during the period with residence and healthcare coverage in Navarra and a variety of information sources were used that virtually rules out losses of follow-up^33^. HIV-related mortality includes more causes than AIDS-mortality, therefore, care should be paid in comparison with studies that analysed AIDS mortality^29,34^. Since important sociodemographic changes happened in the study population, time-trend analyses were adjusted not only by sex and age, but also by country of birth and transmission category to maintain the comparability over time. The impact of direct-acting antiviral therapy of HCV infection in mortality could not be detected since this therapy was just being introduced in the last study period.

Only the main cause of death was considered, but not other relevant causes that could also contribute. These results are conditioned by the HIV epidemiology in Spain, where most HIV infections were acquired by risk practices related with injecting drug use. Undiagnosed HIV infections and mortality that could occur before diagnosis were not represented in this study.

## Conclusions

Advances in the antiretroviral therapy and improvements in the management of HIV-infected patients have coincided with a progressive decline in HIV-related mortality in a population-based cohort of HIV-infected persons, mainly among those who had had diagnosis of AIDS-defined events, while non-HIV-related mortality remained stable. Despite the wide availability of antiretroviral therapies in Spain, HIV infection was still associated to important excess mortality, overall and by many specific non-HIV-related causes. These causes of death have surpassed HIV-related causes, and this trend probably will increase due to the progressive aging of the HIV infected population. Since antiretroviral therapy does not seem enough to correct the excess mortality, prevention of HIV infections in the population and promotion of healthy life styles in HIV-infected people must be a priority.

## Methods

### Design and population

This study analysed the population-based dynamic cohort of adults between 25 and 74 years old diagnosed with HIV infection and residing in Navarra, a northern Spanish region of 650,000 inhabitants approximately. The resident population in the region had easy and free access to medical care and to the HIV test in primary healthcare, specialized outpatient consultations and hospitals. Combined antiretroviral therapies have been available free of charge for the patient in 3 HIV-centres during the study period. HCV antiviral treatments have been offered to HCV co-infected patients and since 2014 direct-acting antiviral therapies were progressively offered to all co-infected patients.

### Information sources and variables

HIV infection reporting is mandatory for healthcare professionals according to the Navarra Decree 383/1997, and patient consent is not required. This study analysed data from the enhanced epidemiological surveillance of HIV-infection that includes all cases confirmed in laboratories of the region since the beginning of the epidemic^35^. It has been prospectively updated by an automatic reporting of all new laboratory-confirmed cases and by the active search of HIV-infected patients attended in medical centres. AIDS-defining events were obtained from the AIDS case reporting system and the hospital discharge database, vital status was updated according the regional mortality register, and changes in the residence were updated from administrative databases of the Navarra Health Service^35^.

The variables considered were sex, birth date, transmission category (history of injecting drug use, men who had sex with men, heterosexual risk exposure, and other risk or unknown), country of birth (Spain or other), date of HIV diagnosis, hepatitis C antibody positive result, date of AIDS diagnosis, date of death and cause of death.

The reference general population consisted of persons residing in Navarra according to the census at the beginning of each year. From the regional mortality register we obtained the date and the underlying cause of death according to the International Classification of Diseases 10th edition^36^, for HIV-infected persons and for the general population. The causes of death were grouped into two main categories, HIV-related deaths (codes B20-B24 and R75) and non-HIV related deaths (all other codes). In the second category we considered separately: non-AIDS defining cancer (codes C00-D48), cardiovascular disease (codes I00-I99), respiratory disease (codes J00-J99), liver disease (codes B15-B19, B70, K73, K74, K769), drug addiction or overdose (codes F11, F16, F18, F19, X41, X42, X44, X45), suicide (codes X60-X84), other external causes (codes V01-Y89, when they were not included in the previous sections), and all other causes.

All data were treated in a strictly confidential manner according to the ethical principles of the Helsinki Declaration of 1964 revised by the World Medical Organization in Fortaleza 2013, and the Spanish and European personal data protection regulations. The study protocol was approved by the Navarra Public Health Authority and the Navarra Ethical Committee for Clinical Research. Patient information was anonymized prior to analysis.

### Statistical analysis

The study period started on first January 1999 for previously diagnosed infections, on the date when HIV infection was diagnosed for new detected cases, and on the date of registration in the Navarra Health Service for previously diagnosed HIV-infected patients who came to reside in the region. The follow-up ceased at the date of death, the date of deregistration as a resident or by 31 December 2018, whichever came first.

Percentages were calculated for categorical variables and were compared by χ^2^ test. Median and interquartile range were obtained for quantitative variables which were compared by the Kruskall Wallis test.

The number of person-years of follow-up was used as the denominator for the calculation of average annual mortality rates for five-year periods. Poisson regression models were used to estimate average annual change in mortality rates, overall, by sex, age, history of injecting drug use, country of birth, diagnosis of an AIDS-defining event, HIV diagnoses before 1997 (year of extended use combined antiretroviral therapy) and groups of causes of death. Rate ratios of annual trend unadjusted and adjusted by sex, age-group (25–44, 45–54 and 55–74 years), transmission category and country of birth were calculated.

The number of observed deaths in the cohort of persons diagnosed with HIV infection was compared with expected mortality of the Navarra general population of the same sex and ten-year group of age. Relative excess mortality was estimated by standardized mortality ratios with their 95% confidence intervals obtained by the exact Mid-P method. Excess mortality rates were calculated as the difference of observed minus expected deaths and divided by the number of PY of follow-up. These analyses were repeated for each five-year period, sex, age group, transmission category and HIV diagnoses before or in or after 1997.

For the last ten-year period (2009–2018) observed mortality in the cohort of HIV-infected persons by causes was compared with the expected mortality estimated from the general population.
